# Recreational cycling provides greater satisfaction and flow in an immersive virtual environment than in real life

**DOI:** 10.1186/s13102-024-00818-4

**Published:** 2024-01-30

**Authors:** Jacek Polechoński, Bartosz Szczechowicz, Jakub Ryśnik, Rajmund Tomik

**Affiliations:** 1 Institute of Sport Sciences, The Jerzy Kukuczka Academy of Physical Education in Katowice, Mikołowska 72A, 40-065 Katowice, Poland; 2 Faculty of Tourism and Leisure, Institute of Entrepreneurship and Management, University of Physical Education in Kraków, Jana Pawła II Avenue 78, 31-571 Kraków, Poland; 3 Department of Health-Related Physical Activity and Tourism, The Jerzy Kukuczka Academy of Physical Education in Katowice, Mikołowska 72A, 40-065 Katowice, Poland

**Keywords:** Virtual reality, Physical activity, Cycling, Satisfaction, Flow

## Abstract

**Background:**

As the state of satisfaction and flow involved in the physical activity (PA) determines future training commitment, it is undoubtedly very important to study the factors influencing the attractiveness of PA. One of such factors is the usage of virtual reality (VR) technology which creates opportunities for its users to practice various forms of PA in a altered way. It is interesting whether PA practiced in a virtual environment can offer higher levels of satisfaction and flow comparing with PA practiced in the real world. Positive answer to this question support the statement that the use of such technology could contribute to the future commitment in PA. Therefore, in order to find out whether PA in VR can be an attractive alternative towards the PA in a real world, the research should be undertaken to verify if the state of satisfaction and flow involved in the practising certain PA in the VR environment could be higher comparing to the levels of pleasure and flow connected with the same PA carried out in the real world.

**Objective:**

The main objective of the study was to assess the level of satisfaction and flow experienced by healthy adults during various cycling conditions: real life (RL), non-immersive and immersive virtual reality (nIVR and IVR). Additionally, questionnaires for assessing satisfaction with PA and flow in RL and VR were also validated in terms of their measurement reliability. The correlation of the results obtained during tests using both measurement tools was also assessed.

**Methods:**

Forty students were studied, including 20 women (age 22.35 ± 2.32 years) and 20 men (age 22.95 ± 2.19 years). The Physical Activity Enjoyment Scale (PACES) was used to evaluate the enjoyment of cycling. Flow state was assessed using The Flow State Scale (FSS).

**Results:**

Based on Friedman’s analysis of variance regarding the results obtained for all the respondents, it can be concluded that the conditions of cycling significantly affect their level of satisfaction (χ2 = 85.61(40;3); *p* < 0.001) and flow (χ2 = 40.52(40;3); *p* < 0.001). The research participants rated cycling the highest in IVR. Based on the calculated Cronbach's alpha coefficients, high measurement reliability of the questionnaires used in nIVR (PACES, α = 0.94; FFS-2, α = 0.86) and IVR (PACES, α = 0.89; FFS-2, α = 0.91). There was also a significant positive moderate correlation between PA satisfaction and user flow.

**Conclusions:**

The research is the first attempt to directly compare the sense of satisfaction and flow when practicing cycling in RL and in nIVR and IVR. The greater attractiveness and higher level of flow during PA in IVR compared to a similar traditional form of PA in RL, found on the basis of the conducted research, should prompt reflection by both those involved in planning and promoting PA, as well as the creators of active video games (AVGs). Due to the great attractiveness of PA in IVR and the constant, dynamic development of immersive information technologies, virtual training may, in the near future, become not only an important supplement to conventional forms of exercise, but perhaps even an alternative solution.

## Background

The dynamic development of information technology in recent years has significantly changed people's ability to manage their free time. This is also true within the context of physical activity (PA). The fascination, especially among young people, with computer games promotes their sedentary behaviour. Paradoxically, however, interest in this technology may contribute to the adoption of newly developed forms of PA, such as active video games (AVGs) or interactive training programmes that can be practiced in virtual reality (VR). The durability and regularity of these behaviours depend on positive user experiences. How people feel while exercising determines their future training commitment [1], and satisfaction from PA is considered an important predictor of exercise adherence [2, 3]. Increasing satisfaction may favour systematic training in the long run. Therefore, it is justified to ask whether the hedonistic experiences of people engaging in recreational PA in VR differ significantly from the experiences arising as a result of analogous activities carried out in natural conditions, which can be summarized in the category of so-called real life (RL). While the intuitive answer to this general question seems to be affirmative, it is interesting to reveal possible similarities and differences at a more in-depth level in the experiences resulting from participation in PA in the two indicated environments: real and virtual. Bearing in mind that conducting an thorough analysis of the indicated issue requires referring to a specific type of PA, the general objective of this article is to compare the hedonistic experiences of young people related to undertaking typically recreational cycling training in natural and VR conditions.

The use of VR in research requires a precise definition of this concept, as the conventional terminology is not always properly defined in literature on the subject [4]. Undoubtedly, VR is an artificially created reality using information technology that can depict elements of both the real world (e.g. computer simulations) and a completely fictional one (e.g. computer games). Depending on the extent to which the user is isolated from the real world when interacting with the virtual environment, a distinction can be made between non-immersive virtual reality (nIVR) and immersive virtual reality (IVR). [5]. In the former, the virtual environment is usually displayed on a standard computer monitor and interaction is limited to using a pointing device, while in the case of the latter, the user is surrounded by a three-dimensional, computer-generated presentation. The person can use his/her own body for sensory-motor interaction with the virtual environment [4]. In such a situation, a person is cut off from visual stimuli of the real world, but instead receives digitally created visual, auditory and even tactile stimuli. IVR presentation is possible through the use of head-mounted displays (HMDs) or room-sized systems called CAVE (C-Automatic Virtual Environment) [6]. The differences between IVR and nIVR are primarily related to the level of immersion. The second significant difference is the use of the user's body, which in IVR becomes the main interface for interaction with the virtual world [7]. In IVR, the user can interact with the virtual environment not only by using a joypad or keyboard, but also by turning his/her head, through eye movements or specially designed controllers that respond to the position and movements of the player's body in a specific space. These features promote greater user involvement, enhance their emotions, while eliminating distracting elements from the outside world [7, 8].

Despite there being a lot of research on users' experiences with VR, there is little information about the differences in the experiences of people using the application in immersive and non-immersive modes [6]. This applies especially to so-called active video games (AVGs) stimulating physical activity and virtual training programmes. Applications inducing a greater sense of presence can increase concentration on the received stimuli that are perceived as belonging to the real world and, as a consequence, should cause high psychophysical arousal [9]. It should be noted that the greatest sense of presence is achieved by fully immersive systems, which is visible both at the level of physiological reactions and in the subjective feelings of users [10]. From the research conducted by Pallavicini et al. [6, 11] and Tan et al. [12] it results that when playing commercial video games in an immersive mode, users experience more intense experiences accompanied by stronger emotional reactions than when using the same applications on a monitor. Therefore, when planning to compare the feelings associated with practicing similar forms of PA in RL and VR, different levels of immersion should be taken into account.

Research conducted in recent years allows to show that AVGs practiced in both non-immersive and immersive VR are positively assessed by players in terms of attractiveness [13–16]. Within the context of user satisfaction, however, both the type of exercise task performed and its intensity are of significance [14]. That is why, when planning to compare the sensations associated with practicing similar forms of AF in nIVR and IVR, it is necessary to ensure that users experience them with a similar exercise load applied.

A high degree of satisfaction with the performed activities is associated with the concept of the so-called flow. This concept describes an optimal mental state in which users are fully engaged in the activity they are performing, experiencing a high level of focus, control and pleasure [17, 18]. Due to the fact that this condition may occur both when practicing various forms of PA in RL and VR, it is worth also examining the level of flow felt while assessing the satisfaction of exercisers.

Considering all the above issues, the main aim of this study is to evaluate the level of satisfaction and flow according to different cycling conditions (RL, nIVR, IVR) among healthy adults. Additionally, questionnaires for assessing satisfaction with PA and flow in real and virtual environments were also validated in terms of their measurement reliability. The correlation of the results obtained during tests using both measurement tools was also assessed. It was hypothetically assumed that satisfaction and the experience of flow significantly depend on the conditions of cycling, and off-road cycling (RL) will be perceived most positively in this respect by the respondents. The goals formulated in this way allowed us to pose the following research questions:

1. Do the different conditions of recreational cycling significantly affect PA-related satisfaction and flow?
2. Are standard questionnaires for assessing satisfaction with PA and flow a reliable measurement tool for cycling in nIVR and IVR for young, physically fit adults?
3. Is there a correlation between satisfaction with PA and the flow of users practicing various forms of cycling in real conditions and in VR?

## Methods

### Participants

In accordance with the adopted inclusion criteria, people who were currently studying at the Academy of Physical Education, healthy and physically fit, able to ride a bicycle and who undertook this type of PA, including riding a cycle ergometer, could participate in the study. The following exclusion criteria were adopted: sensitivity to flashing light or image patterns that may appear in programs and video games, suffering from epilepsy, having symptoms of motion sickness or balance disorders, and having previously used the tested application. Ultimately, the research covered 40 volunteers who met the indicated conditions—students of the Academyy of Physical Education (age 22.65 ± 2.25 years), including 20 women (age 22.35 ± 2.32 years) and 20 men (age 22.95 ± 2.19 years). The respondents' previous experience with IVR technology was varied: 18 people (45%) had never used it, 14 (35%) had used it very rarely, 4 (10%) had used it rarely, 3 (7.5%) often, and only 1 (2.5%) declared having used IVR very often.

The study was conducted according to the guidelines of the 1964 Declaration of Helsinki and it was reviewed and approved by the Research Ethics Committee of the Jerzy Kukuczka Academy of Physical Education in Katowice (protocol 9/2018). All participants took part in the study voluntarily and could discontinue their participation at any time.

### Procedures and research methods

The research was carried out at the Jerzy Kukuczka Academy of Physical Education in Katowice at a certified Laboratory of Research on Pro-Health Physical Activity (PN-EN ISO 9001:2015, certificate validity: 7.12.2021–16.12.2024) between June-July 2023.

It was assumed that the research would be carried out in relation to "bicycle cycling"—as one of the most accessible and popular active forms of spending free time in society. PA was considered in two forms: traditional—carried out using a standard bicycle, and stationary—using a cycle ergometer. While typical riding referred to activities performed in natural terrain, stationary riding was considered in relation to three different, varying conditions: as riding without any visualisations and as riding in an artificially created environment in the nIVR and IVR variants. In total, four research variants were considered: (1) Riding a standard bike in natural conditions, (2) Riding a stationary bike without any visualisations, (3) Riding a stationary bike in nIVR, (4) Riding a stationary bike in IVR.

Following the principle of research process rationality, it was assumed that the first two activities are so common and—as verified at the sample selection stage—known to the research participants that they can assess them reliably using a questionnaire, without having to undertake them during the research. As for the other two activities, it was found that they were so unique and poorly recognised by the research participants that completing the related questionnaires must be preceded by specially organised and staged cycling sessions. Therefore, the essential element of the research procedure was, in practice, two sessions of activity for the studied participants:

- “Session 1”: riding a stationary bicycle in nIVR, during which the image of the virtual world was displayed on a screen located in front of the subject;
- “Session 2”: riding a stationary bicycle in IVR using VR goggles.

At the beginning of the study, the participants completed a form containing questions allowing to collect data on their previous contact with VR and the frequency of using this technology. They then completed a total of four questionnaires:

- before starting the activity session, they filled out "Questionnaire No. 1" (concerning riding a standard bicycle in natural conditions) and "Questionnaire No. 2" (concerning riding a stationary bicycle without any visualisations);
- after completing "Session 1", they filled out "Questionnaire No. 3" (concerning stationary cycling in nIVR);
- after completing "Session 2", they filled out "Questionnaire No. 4" (concerning stationary cycling in IVR).

All questionnaires contained two identical sets of questions regarding, respectively: the level of satisfaction with PA and the state of flow related to PA. The difference between them concerned only the type of activity for which research participants provided answers.

As a set of questions regarding level of satisfaction, the Physical Activity Enjoyment Scale (PACES) – short version, was used [19]. This research tool consists of 8 statements, each of which indicates a different aspect of broadly understood "pleasure" concerning PA: “I find it pleasurable”, “It’s a lot of fun”, “It’s very pleasant”, “It’s very invigorating”, “It’s very gratifying”, “It’s very exhilarating”, “It’s very stimulating”, and “It’s very refreshing”. Each statement was assigned a 7-point Likert scale (1—"strongly disagree", 7—"strongly agree"). The research participants' task was to mark, for each statement, the value that best reflected their feelings about the activity considered in a given situation. The result was the average of the points obtained in all questions.

The Flow State Scale (FSS), also in its short version, was adopted to identify "flow" state [20]. This research tool comprises 9 statements, each of which corresponds to one of the dimensions of the flow state arising during PA: *Challenge-Skill Balance:* “I feel I am competent enough to meet the high demands of the situation”; *Action-Awareness Merging:* “I do things spontaneously and automatically without having to think”; *Clear Goals:* “I have a strong sense of what I want to do”; *Unambiguous Feedback:* “I have a good idea while I am performing about how well I am doing”; *Concentration on Task at Hand:* “I am completely focused on the task at hand”; *Sense of Control:* “I have a feeling of total control over what I am doing”; *Loss self-consciousness:*”I am not worried about what others may be thinking of me”; *Transformation of Time:* “The way time passes seems to be different from normal”; *Autotelic Experience:* “The experience is extremely rewarding”. Each statement is assigned a 5-point Likert scale (1—"strongly disagree", 5—"strongly agree"). The respondents' task was to indicate, for each statement, the value that best reflected their feelings about the activity considered in a given situation. The result was the average of the points obtained for all questions.

The activity sessions of the subjects were carried out at a purposefully organised research station. The basic element was a cycloergometer (Kettler—Ergo C10) equipped with an external cadence sensor (Wahoo Cadence), attached to the crank mechanism. Before beginning the study, the height of the cycloergometer seat and the strap securing the VR goggles were adjusted. It was assumed that PA would be implemented by research participants at a moderate level. This intensity of exercise is associated with recreational physical activity, and according to the guidelines of the World Health Organization (WHO), it is sufficient to obtain health benefits [21]. The maximum heart rate (HR_max_) was calculated for each examined person using the formula: 208–0.7 × age [22]. On the basis of the classification proposed by American College of Sport Medicine [23], it was determined what heart rate level would correspond to moderate intensity, i.e. meaning it would be within the range of 64–77% HR_max_.

In the case of both riding sessions on the cycloergometer, the training mode available in this device was activated with the load automatically controlled depending on the parameters of the subject's heart rate, which was continuously monitored using a heart rate sensor (Polar H10) placed on the chest and cooperating with the cycloergometer via Bluetooth. The cycloergometer automatically changed the load so that the subject's heart rate remained within the zone of 64–77% HR_max_, i.e. the PA intensity was at a moderate level. Due to this, each of the research participants focused their attention on their experiences with PA in nIVR and VR with a similar exercise load. In this way, a comparable exercise environment was created for all subjects, not only within the context of the presented image and type of PA, but also regarding the intensity of physical exercise. During the training session, the subjects could monitor heart rate both in nIVR on the cycle ergometer screen and in IVR on the goggle display.

**Fig. 1:**
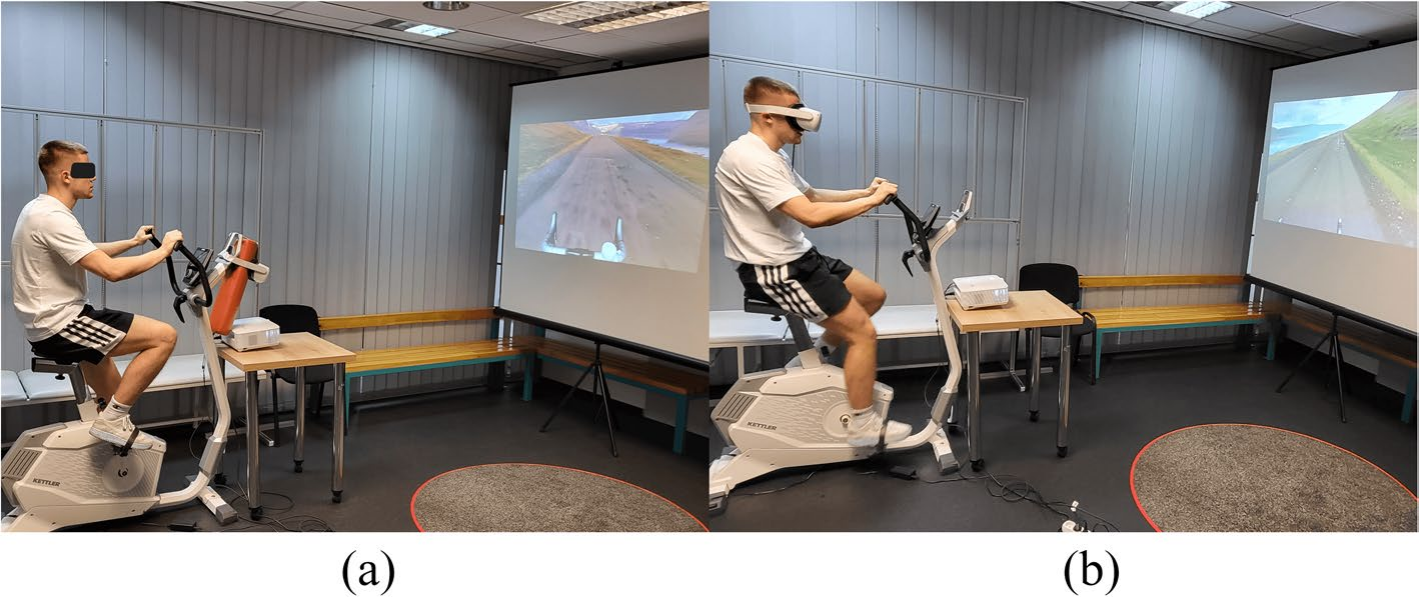
Research station. **a** “Session 1” – riding stationary bicycle in nIVR; **b** “Session 2” – riding stationary bicycle in IVR

For the purposes of "Session 1" (nIVR), the view of the artificially generated reality was displayed via a multimedia projector on a projection screen located in front of the subject at a distance of 250 cm; image diagonal = 200 cm (Fig. 1a). The research participant, riding a stationary bike, "overcame" the route, observing the changing surroundings, but at the same time, receiving visual and auditory stimuli from the real environment. During "Session 2" (IVR), the subject immersed him/herself in an artificially generated reality through wearing Oculus Quest 2 VR goggles (Fig. 1b). In this manner, the research participant was cut off from visual and auditory stimuli coming from the real environment, and therefore, focused only on the undertaken activity and the virtual environment.

Both cycle ergometer riding sessions were conducted using the VZfit application (https://www.vzfit.com/). The application allows users to create routes and "travel" along them to the extent that photos are made available via the Google Street View service. The application processes photos in such a way that the virtual reality dynamically created on their basis simulates the changing image that a cyclist can see while moving along a given route. VZfit allows to move around locations and their assigned routes thanks to the application's cooperation with the cadence sensor located on the crank mechanism of the cycloergometer.

**Fig. 2:**
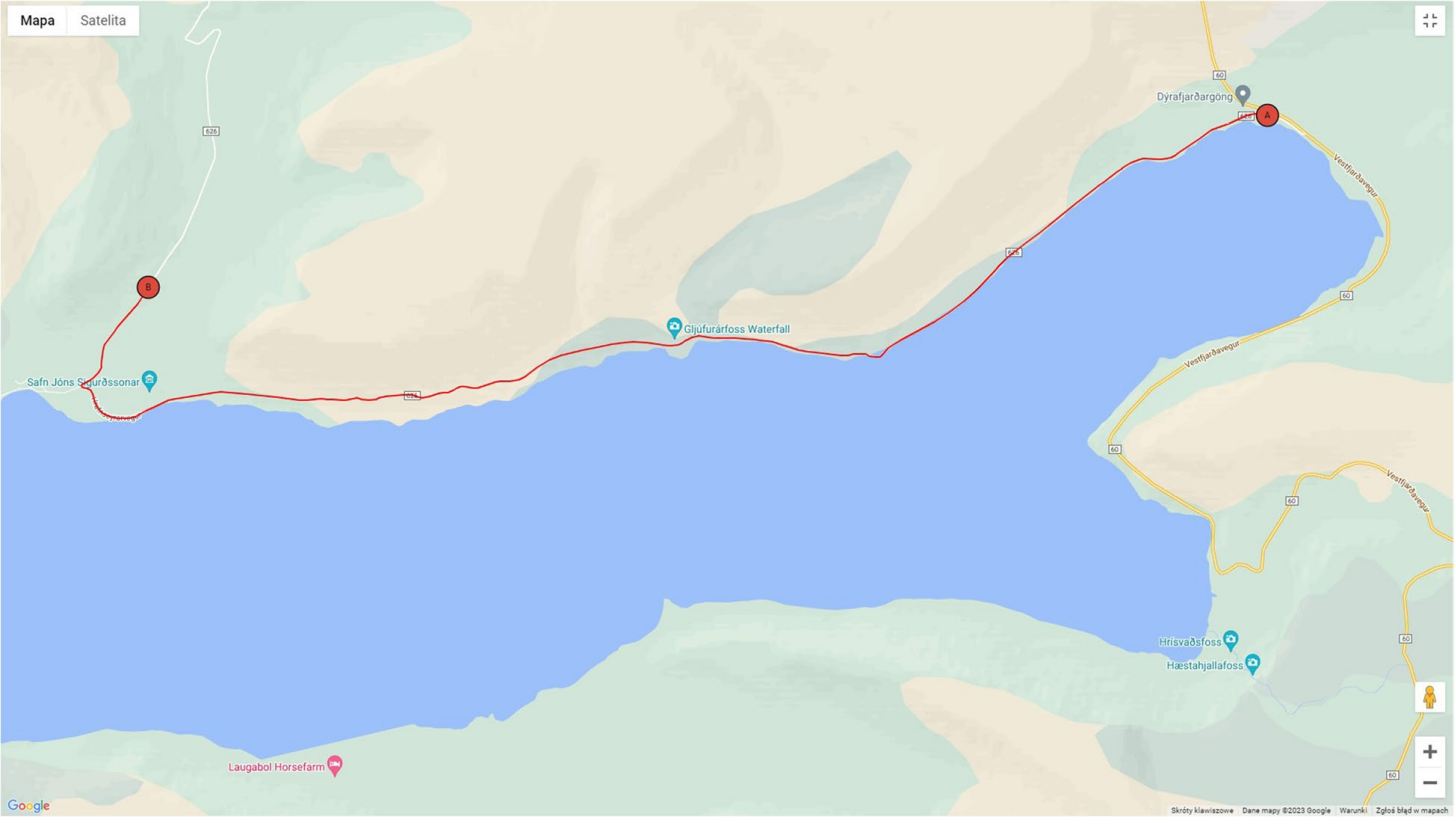
Map of route”covered” by study participants. Source: Screenshot from account of VZfit application user (https://www.vzfit.com)

**Fig. 3:**
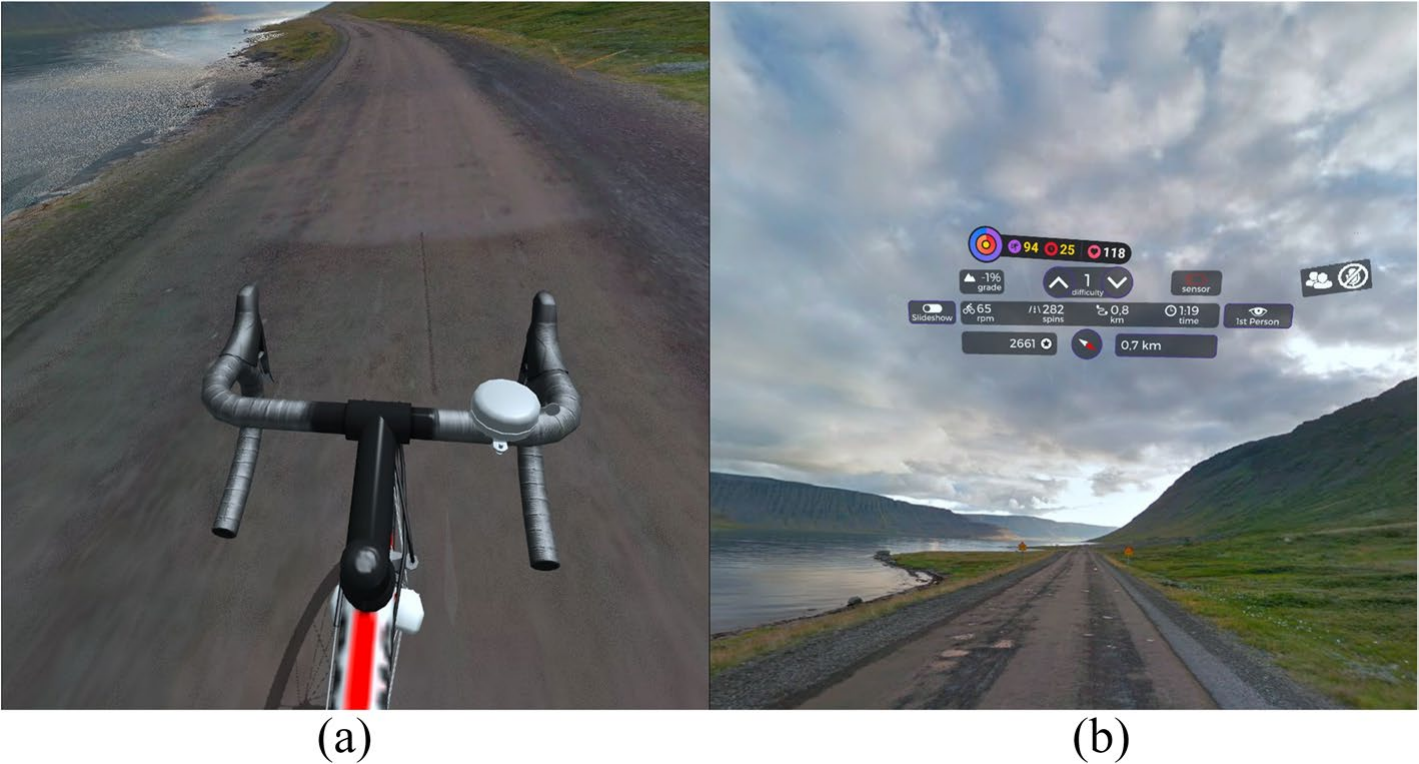
View of route covered by study participants. **a **standard – through VR goggles”forward”; **b **after looking “up” in VR goggles (visible route parameters and heart rate); source: Screenshot from Oculus Quest 2 goggles using VZfit application

After performing tests and test rides, it was decided to choose a route that allows for at least a dozen or so minutes of riding at a moderate pace, in an area without buildings (the processing of photos of buildings in VZfit was carried out in an unnatural way), and which does not include large uphill or downhill slopes, after which the user would expect a change in load (the load was controlled only by the subject's heart rate). The route chosen was 14.8-km long and ran along the coast of Iceland on road 625, passing the Gljufurarfoss waterfall. It was created in the VZfit application by user KenOhBee and is available under the name Iceland Sunset Ride (Figs. 2 and 3).

The standard control method in the VZfit application was selected, which involves automatic change of riding direction in accordance with the designated route. Gamification elements have been disabled, such as the company of a trainer in the form of a bot or other cyclists—application users, or collecting virtual coins. These additions would be an element not usually present during cycling. However, natural environmental effects that were considered authentic (rain, flying birds) were included.

It should be added that subsequent subjects participated in the activity sessions in a different order, i.e. every second person first started riding in nIVR conditions, and then in IVR, and every second person—first in IVR and then in nIVR. In this way, attempts were made to eliminate any possible impact on the research results of the order in which the sessions were to be conducted. Each training session lasted 10 min. There was a interval for rest totalling several minutes between the sessions, allowing the subjects' heart rate to stabilise at resting level. The estimated time to complete the study for one person was 1 h.

### Statistical methods

Statistical calculations were performed using Statistica and SPSS software. Measurement data were analysed using basic descriptive statistics. Arithmetic means and standard deviations were calculated. The Shapiro–Wilk test was used to assess the normality of distribution. The correlations between various cycling conditions and the level of satisfaction and flow of the respondents were estimated using Friedman's ANOVA, for which the effect size was calculated on the basis of Kendall's W coefficient. In order to make intra-group comparisons, Friedman’s test was supplemented with Dunn's post-hoc tests using the Bonferroni correction. The non-parametric Mann–Whitney U test was applied for comparisons between women and men. The reliability of the questionnaires used in the research was assessed by analysing the internal consistency of the test via Cronbach's alpha coefficient (α). The correlation between the PACES and FSS questionnaire results was estimated using Spearman's correlation coefficient.

## Results

### Correlations between cycling conditions and satisfaction of study participants

**Fig. 4:**
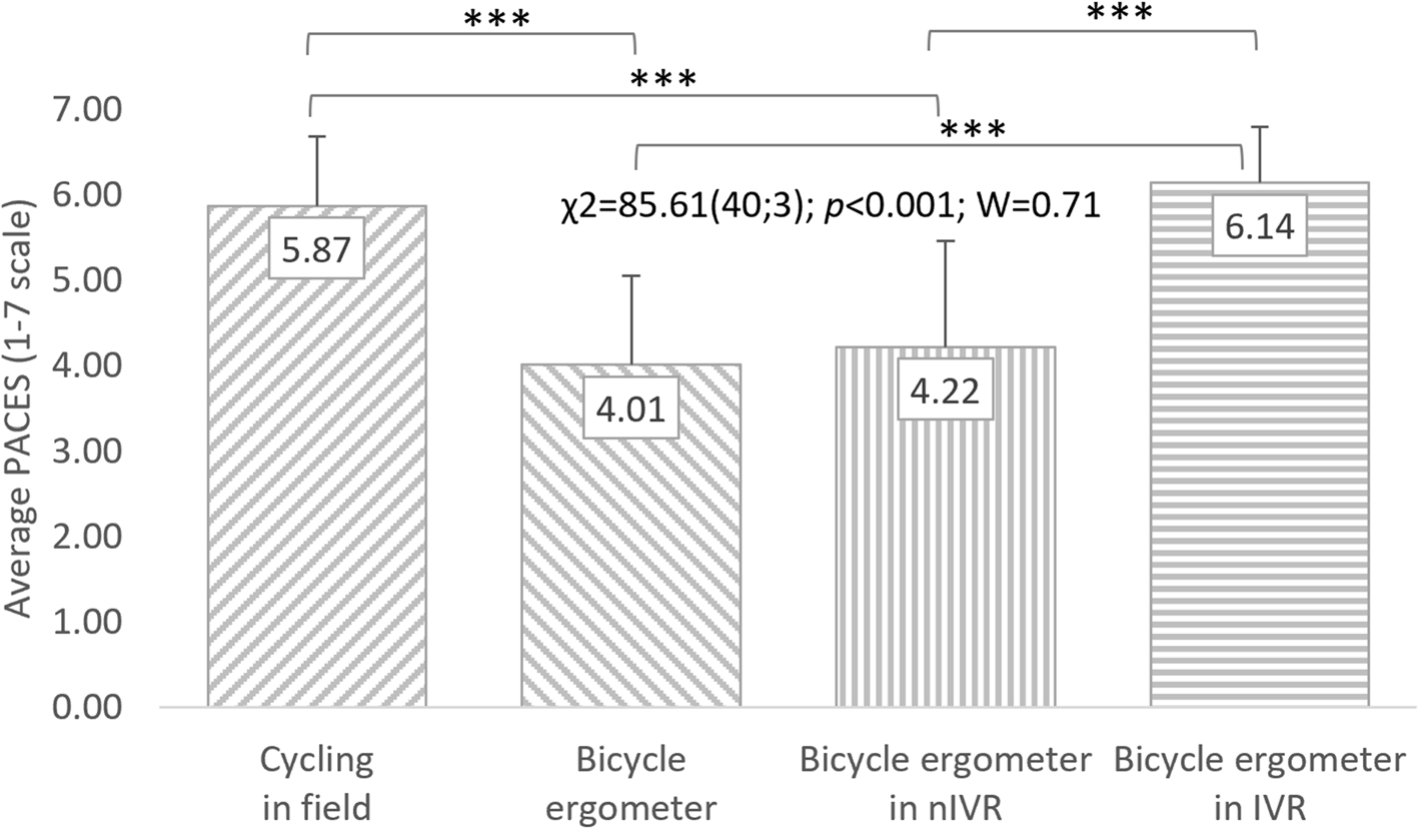
Correlations between cycling conditions and students’ satisfaction (total). χ^2^: test statistics of Friedman’s test; *p*: *p* value; W: Kendall’s coefficient; ***p < 0.001

Based on Friedman’s analysis of variance regarding the PACES results of all respondents, it can be concluded that the conditions of cycling significantly affect level of satisfaction (χ^2^ = 85.61(40;3); *p* < 0.001; W = 0.71). The study participants rated cycling in IVR highly (6.14 ± 0.65 points) and in the field (5.87 ± 0.82 points). Less attractive, in the opinion of the subjects, was cycling using a cycle ergometer in nIVR (4.22 ± 1.24 points) and in conditions of no exposure to VR (4.01 ± 1.05 points). Extended post-hoc analyses allow to indicate the occurrence of statistically significant differences (*p* < 0.001) in the respondents' perception of satisfaction with PA while cycling in IVR and exercise on a cycle ergometer using nIVR and without VR. Significant differences were also noted when comparing mean PACES scores for outdoor cycling with PA on a cycle ergometer using nIVR and without VR (Fig. 4).

**Fig. 5:**
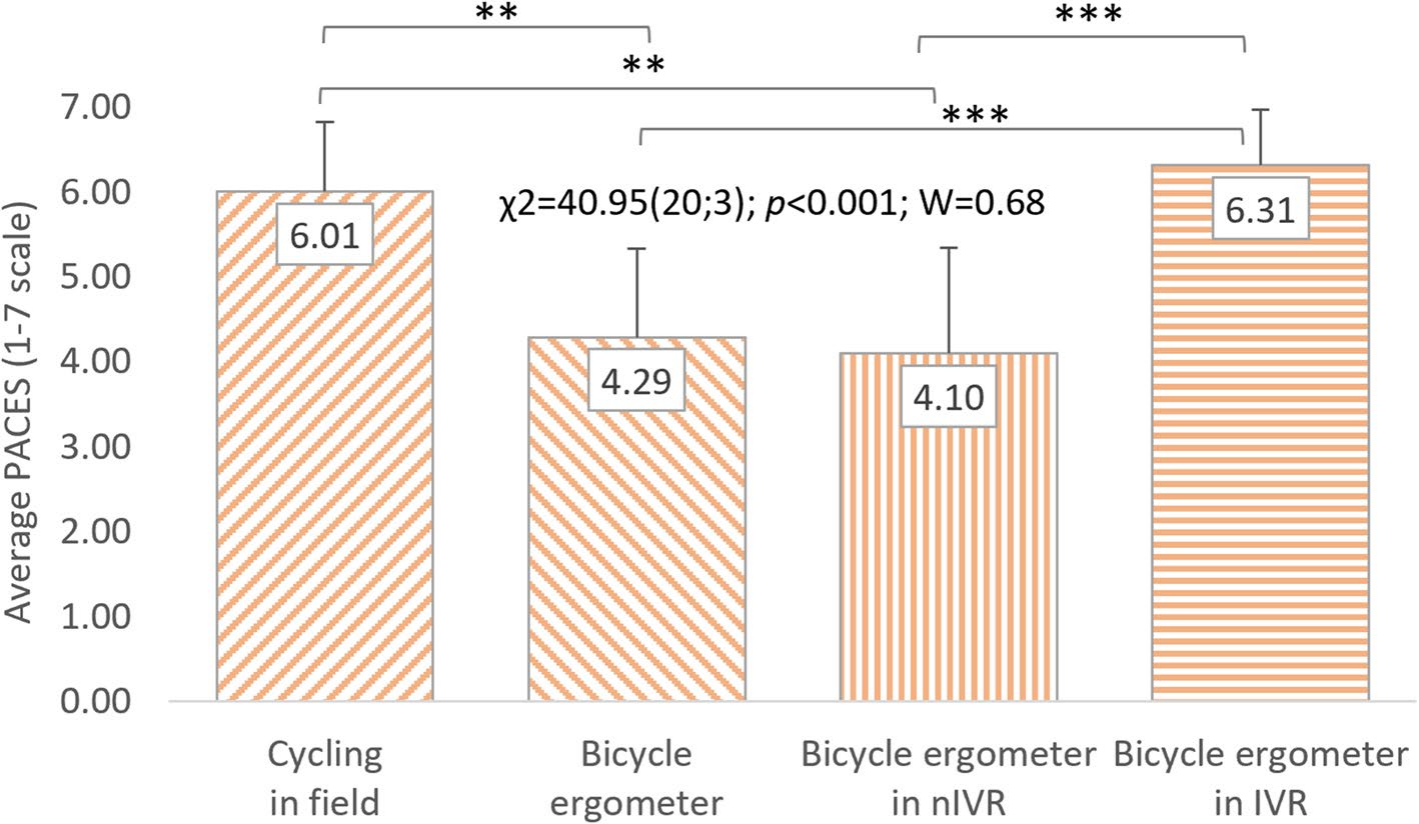
Correlations between cycling conditions and students’ satisfaction (women). χ^2^: test statistic for Friedman’s test; *p*: *p* value; W: Kendall’s coefficient; **p < 0.01; ***p < 0.001

**Fig. 6:**
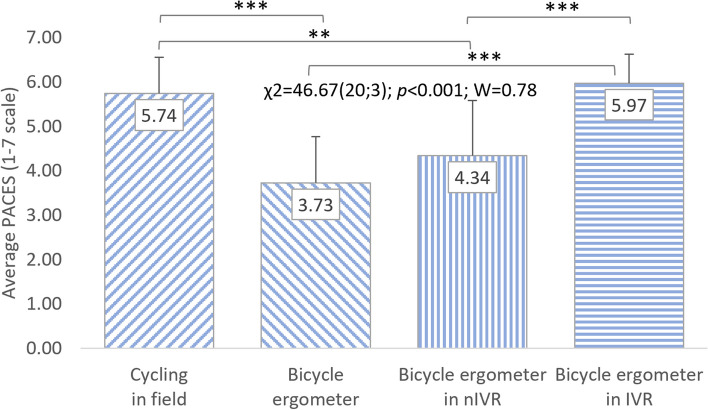
Correlations between cycling conditions and students’ satisfaction (men). χ^2^: test statistic for Friedman’s test; *p*: *p* value; W: Kendall’s coefficient; **p < 0.01; ***p < 0.001

A significant impact of cycling conditions on the level of satisfaction with PA is also visible in the analyses of variance regarding the PACES questionnaire results conducted separately for women (χ^2^ = 40.95(20;3); *p* < 0.001; W = 0.68) and men (χ^2^ = 46.67(20;3); *p* < 0.001; W = 0.78). Both of these groups rated cycling the highest in IVR (6.31 ± 0.56 points; 5.97 ± 0.71 points) and in the field (6.01 ± 0.63 points; 5.74 ± 0.97). However, satisfaction with practicing the other two forms of cycling was rated much lower. Women were more satisfied with cycle ergometer riding without exposure to VR (4.29 ± 0.83 points) than with nIVR (4.10 ± 1.32 points). Men, on the other hand, rated the attractiveness of PA using nIVR higher (4.34 ± 1.17 points) than training on a cycle ergometer without VR (3.73 ± 1.18 points). In the case of both women and men, the statistically significant differences in the perception of satisfaction with PA depending on the conditions of cycling, shown on the basis of post-hoc tests, occurred in similar situations as for all respondents (Figs. 5 and 6). However, no statistically significant differences in PACES results were observed between women and men (Table 1).

**Table 1:** Differences in perception of satisfaction with cycling in various conditions by women and men

Cycling conditions	Women ($\overline{{\text{x}} }$ ± SD)	Men ($\overline{{\text{x}} }$ ± SD)	d	U	*p*
Cycling in field	6.01 ± 0.63	5.74 ± 0.97	0.27	171.00	0.441
Bicycle ergometer	4.29 ± 0.83	3.73 ± 1.18	0.56	147.50	0.160
Bicycle ergometer in nIVR	4.10 ± 1.32	4.34 ± 1.17	-0.24	189.50	0.786
Bicycle ergometer in IVR	6.31 ± 0.56	5.97 ± 0.71	0.34	147.00	0.154

$\overline{{\text{x}} }$ arithmetic mean, *SD* standard deviation; d: difference, *U* Mann–Whitney U test value; *p*: *p* value

### Assessment of PACES reliability in group of study participants

According to Chen et al. [24] PACES is a reliable and valid tool for measuring satisfaction with PA, with high test–retest repeatability (r = 0.76) and internal consistency (α = 0.82 to 0.88). In order to validate PACES in the group of subjects, Cronbach's alpha was calculated for the questionnaires regarding all cycling conditions. The highest values of the α index were recorded in the case of assessing satisfaction with practicing cycling in nIVR (α = 0.94) and in the field (α = 0.93). Slightly lower values were recorded for cycling on a cycle ergometer in VR (α = 0.89) and without exposure of the subject to VR (α = 0.89). Therefore, the results were high because an acceptable alpha value would be within the range of 0.70 and 0.95 [25].

### Correlations between cycling conditions and flow of study participants

**Fig. 7:**
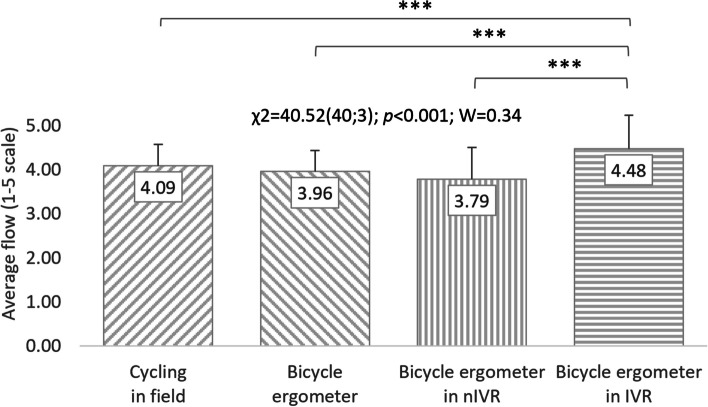
Correlations between cycling conditions and students’ flow level (total). χ^2^: test statistic for Friedman’s test; *p*: *p* value; W: Kendall’s coefficient; ***p < 0.001

Friedman’s analysis of variance regarding the results obtained in the FSS questionnaire of all respondents (in total) allows to indicate that the conditions of cycling significantly affect the respondents' flow (χ^2^ = 40.52(40;3); *p* < 0.001; W = 0.34). The respondents rated cycling in IVR the highest (4.48 ± 0.76 points). Within the context of perceived flow, students rated outdoor cycling at 4.09 ± 0.48 points, cycling using a cycle ergometer at 3.96 ± 0.47 points, and in nIVR at 3.79 ± 0.72 points. Post-hoc analyses allow to show that the respondents' feeling of flow while riding a bicycle in IVR differs significantly (*p* < 0.001) from the feeling of this state in other considered conditions (Fig. 7).

**Fig. 8:**
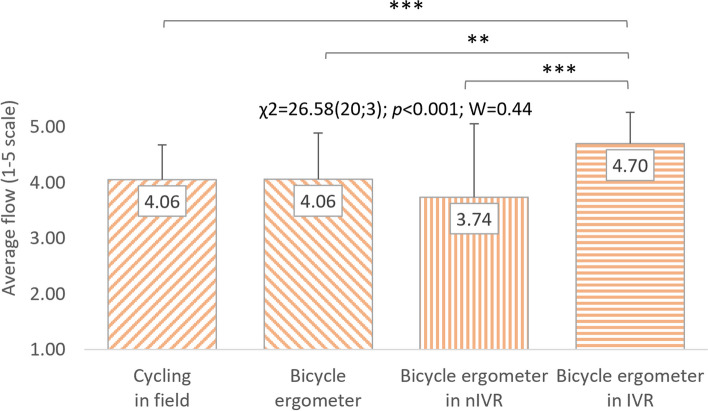
Correlations between cycling conditions and students’ flow level (women). χ^2^: test statistic for Friedman’s test; *p*: *p* value; W: Kendall’s coefficient; **p < 0.01; ***p < 0.001

**Fig. 9:**
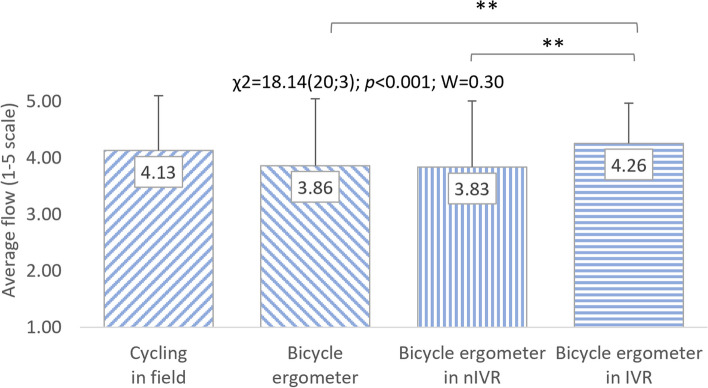
Correlations between cycling conditions and students’ flow level (men). χ^2^: test statistic for Friedman’s test; *p*: *p* value; W: Kendall’s coefficient; **p < 0.01

Friedman's analysis of variance regarding the FSS questionnaire results was also performed separately for representatives of both sexes. A significant impact of cycling conditions on the flow level was visible both in women (χ^2^ = 26.58(20;3); *p* < 0.001; W = 0.44) and in men (χ^2^ = 18.14(20;3); *p* < 0.001; W = 0.30). Similarly to all respondents, the highest level of flow in women (4.70 ± 0.85 points) and men (4.26 ± 0.60 points) was characteristic of cycling in IVR. Women rated cycling outdoors (4.06 ± 0.64 points) and on a cycle ergometer (4.06 ± 0.31 points) equally, and cycling in nIVR was assessed the lowest (3.74 ± 0.85 points). In terms of feeling flow, men ranked PA accompanying outdoor cycling in second place (4.13 ± 0.51 points), followed by typical cycle ergometer riding (3.86 ± 0.59 points) and stationary cycling in nIVR (3.83 ± 0.58 points). Post-hoc analysis allows to demonstrate that the respondents' feeling of flow while riding a bike in IVR differs significantly (*p* < 0.01 or *p* < 0.001) from the feeling of this state in the other three conditions. In men, significant differences in FSS were noted only when comparing respondents' assessment results regarding cycling in IVR with PA on a cycle ergometer without exposure to VR (*p* < 0.01) and in nIVR (*p* < 0.01) (Figs. 8 and 9). No statistically significant differences in FSS scores were observed between the studied women and men (Table 2).

**Table 2:** Differences in perception of flow while cycling in various conditions by women and men

Cycling conditions	Women ($\overline{{\text{x}} }$ ± SD)	Men ($\overline{{\text{x}} }$ ± SD)	d	U	*p*
Cycling in field	4.06 ± 0.46	4.13 ± 0.51	-0.07	180.00	0.60
Bicycle ergometer	4.06 ± 0.31	3.86 ± 0.59	0.20	150.00	0.18
Bicycle ergometer in nIVR	3.74 ± 0.85	3.83 ± 0.58	-0.09	194.50	0.89
Bicycle ergometer in IVR	4.70 ± 0.85	4.26 ± 0.60	0.44	146.50	0.15

$\overline{{\text{x}} }$ arithmetic mean, *SD* standard deviation; d: difference, *U* Mann–Whitney U test value; *p*: *p* value

### Assessment of FSS questionnaire reliability in the group of study participants

According to Jackson et al. [20] the short version of the FSS is a reliable tool allowing to measure flow state and compare it with other psychometric measurements. The estimated value of the α coefficient for this version of the questionnaire was at the level of 0.77–0.78 [20]. (α = 0,60). In our research, Cronbach's alpha of the FSS questionnaire for all respondents was slightly below the acceptable value in the case of students' assessment of cycling in the field (α = 0.69) and traditional training on a cycle ergometer (α = 0.60). However, Kline [26] suggested that this value can be as low as 0.60 for psychological constructs [27]. High values of the α index characterised this questionnaire in relation to the assessment of flow related to cycling in nIVR (α = 0.86) and in IVR (α = 0.91).

### Correlations determined for PACES and FSS results

Based on Spearman's correlation analyses, which were performed for the entire study population, it can be concluded that there are significant correlations between PACES and FSS results. Their level depends on what cycling conditions the study concerned. The highest correlation coefficient (*p* = 0.51) was recorded for the trials related to outdoor cycling. Slightly smaller correlations were noted in the case of assessment regarding cycling in nIVR (*p* = 0.49), on a cycle ergometer (*p* = 0.43) and in IVR (*p* = 0.42). Adopting the classification proposed by Dancey and Reidy [28], the noted correlations should be considered moderate.

## Discussion

### Cycling in various conditions and satisfaction of study participants

Based on the analysis of the research results, it can be clearly stated that cycling conditions have significant impact on satisfaction felt while practicing cycling. Somewhat surprisingly, the most pleasant form of exercise turned out to be PA in IVR which, in this respect, was slightly greater than even physical exercise undertaken on a bicycle in the field—although the noted difference was not statistically significant. Before beginning the experiment, it was assumed that healthy and physically fit Academy of Physical Education students (future promoters of health and PA) would most prefer outdoor cycling. Exercises performed on a cycle ergometer in nIVR and without image projection were rated significantly lower, with women deriving slightly greater pleasure from traditional cycle ergometer riding, while men declared greater satisfaction when exercising in a non-immersive virtual environment. The high values of Cronbach's alpha obtained for questionnaires regarding all cycling conditions confirm the results of the conducted research.

Cycling in IVR and on a traditional cycle ergometer was compared in the research conducted by Zeng et al. [29]. The study group consisted of 12 healthy college students. The load while riding was adjusted so that the heart rate remained within the range of 65–85% of maximum heart rate. The authors assessed blood pressure (BP), rating of perceived exertion (RPE), self-efficacy and enjoyment, which was assessed using a 5-point Likert scale and a survey containing 5 questions. Although there were no significant differences in pre- and post-exercise systolic or diastolic BP changes between the two exercise sessions, participants reported significantly higher RPE during traditional ergometer cycling compared to the IVR cycling session. According to the authors, this is likely attributable to the enjoyable nature of the IVR experience, which could potentially promote user motivation to exercise while having implications for PA promotion. However, the level of students' self-efficacy and satisfaction was significantly higher in IVR than in the case of traditional cycle ergometer riding, which is consistent with the results obtained in the present study, which, however, was carried out applying a different tool and on a different scale.

Bird et al. assessed satisfaction derived from riding a cycle ergometer under exposure to various audiovisual stimuli [30]. The authors studied 18 young adults using the long version of PACES [31]. The studied subjects performed PA at the ventilatory threshold on a stationary bike in five experimental conditions (music, video, music-video, 360-degree video, and 360-degree video with music) and one control condition (no music or video). In the music condition, participants were exposed to the 10-min playlist while in a visually sterile environment. In the video condition, participants were exposed to the cycling video on a laptop. In the music and video condition, participants viewed the cycling video on a laptop and listened to the 10-min music playlist. In the 360-degree video condition, participants were subjected to the cycling video using the VR HMD. The 360-degree video with music condition entailed viewing the cycling video through the VR HMD while being exposed to the 10-min music playlist. In the control condition, participants did not listen to music or watch any videos. The 360-degree video with music condition elicited the highest ratings of perceived enjoyment. It is true that audiovisual stimuli can enhance satisfaction connected with exercise. This fact is supported in previous works. [32, 33].

Various indoor cycling conditions in terms of enjoyment were also assessed by McDonough et al. [34]. The authors compared three different stationary cycling sessions in a group of 49 students. The first session took place in IVR using a VR HMD working with the PlayStation 4 VR console and AVG; during the second session, the subjects used an application supported by Xbox 360, while the third was a traditional exercise bike ride. The authors' analysis of variance showed significant differences in users' perceived enjoyment of PA between the three cycling sessions. The study participants experienced significantly greater enjoyment from cycling in IVR compared to the other two cycling sessions, which could be considered consistent with the results of the current study. It should be noted, however, that the experiences from sessions No. 1 and No. 2 differed not only in immersion, but also in the plot, because they were implemented using different AVGs, while in the present research, the scenery was the same for IVR and nIVR.

IVR also allows to practice other forms of PA related to locomotion. Recently, treadmills that permit walking or running in a virtual environment have become popular. The first attempts are being made to analyse locomotion activity on treadmills in IVR [35–38]. Similar to cycling, there are studies in which the pleasure associated with this type of PA has been examined. Plante et al. [39] compared the pleasure of walking outdoors with walking the same route (video recording) on a treadmill in nIVR and watching this recording in a seated position. The research participants comprised 112 psychology students. Using the long version of PACES, the authors showed that an actual nature walk was more pleasant than an nIVR projection combined with walking on a treadmill and "moving" while sitting, with the latter rated as the least pleasant. Similar studies in a group of 26 healthy adults using IVR were conducted by Calogiuri et al. [40]. The authors found that actual walking outdoors was significantly more enjoyable than walking on a treadmill in VR, as well as controlling VR without walking. However, the study results could have been influenced by poor postural control in IVR and the "cybersickness" occurring in the subjects.

There is no doubt that, apart from the degree of immersion, there are more factors that influence the feeling of pleasure while riding a bike in a virtual environment. For example, the virtual scenery and cycling infrastructure in which the user travels are important. From the research conducted by Bialkova et al. [41], it results that users' exposure to virtual green landscapes contributed to increasing the attractiveness of cycling experiences. The social aspect is also important, e.g. cooperation between players. In the research carried out by Høeg et al. [42], conducted among a group of older adults, it is indicated that users rated the pleasure of riding a virtual tandem bike together very highly. Almost all participants stated that they would like to participate in the ride again if given the opportunity. Cognitive factors may also be important, such as the perception of distance and speed, which play a significant role when it comes to exercise capacity and training effectiveness. There are studies in which novel cycling training systems are applied in a virtual environment to intentionally manipulate movement speed [43]. The level of satisfaction of video game users may also be influenced by: the possibility of competition and the game mode (single/multiplayer) [44], the type of VR-compatible trainers used [14], the game plot [15] and playability [45]. Undoubtedly, the pleasure of practicing PA in VR may also depend on the quality of the graphics and the choice of route.

Analysing the factors that influence satisfaction with performing PA in IVR, those that may reduce users' pleasure and cause them discomfort should also be mentioned. Of chief importance among them is cybersickness, which is associated with unpleasant physiological consequences of users' exposure to IVR. It is characterised by symptoms such as nausea and dizziness [46]. From the experiments conducted by Yildirim [46], it results that games in IVR cause a higher level of cybersickness compared to traditional games on desktop computers—which should be taken into account when comparing satisfaction with PA in IVR and nIVR. However, according to the reports of the participants in this study, such a situation did not occur. None of the respondents reported poor health or symptoms of cybersickness.

The high assessment of attractiveness regarding cycling using the VZFit application in IVR shown in our research and high satisfaction with PA in an immersive virtual environment confirmed by the authors of the above-mentioned research allows to indicate that IVR training programmes should be taken seriously, because in the near future, they may become not only a PA supplement, but an alternative to traditional forms of PA. It should be borne in mind that IVR technology is constantly and very dynamically developing and improving, which, combined with properly selected trainers, should result in the improved comfort and positive experiences of users when using the application. Due to this, the enjoyment of PA is considered an important predictor of exercise adherence [2, 3], while increasing satisfaction may potentially promote systematic training in the long run. How exercisers feel during PA determines their future involvement in exercise [1]. In accordance with the present research, it can therefore be assumed that people may be more willing to participate in exercises if they take place in IVR.

### Cycling in various conditions and flow of study participants

The results of the conducted research clearly indicate that cycling conditions influence the flow felt by the subjects, similarly to the sense of satisfaction. The highest flow occurred during cycling in IVR, and its level for all respondents and for men was significantly higher than that recorded in the three other cycling conditions, in which flow was at a similar level. When analysing the group of women, significant differences in the results were noted only between IVR and nIVR, as well as between IVR and traditional cycle ergometer riding without image projection. The phenomenon of the most intensely felt flow in IVR by research participants can be explained more easily than in the case of satisfaction. To experience flow at a high level, it is necessary to experience total absorption in the activity currently being performed [47], which is probably facilitated by being in IVR, when a person is cut off from the surrounding visual and auditory stimuli of the real world and can fully focus on the sensations received from the artificially created reality.

The reliability of the questionnaires conducted regarding flow while riding a cycle ergometer regarding IVR and nIVR is confirmed by a high level of Cronbach's alpha values. However, the internal consistency of the FSS may raise some doubts in relation to users' assessment of the other two forms of cycling. In the case of outdoor cycling and traditional training on a cycle ergometer, Cronbach's alpha in the case of the FSS questionnaire for all respondents was slightly below the limit of acceptability. Therefore, the analysis of the results of these questionnaires should be approached with some caution. The lower values of the α coefficient in relation to both cycling conditions apart from VR may be due to the fact that the FSS questionnaire referred to users' previous experiences, i.e. this assessment was retrospective.

Examining flow experience is important both within the context of undertaking PA and sports, as well as in the process of creating computer applications, with particular emphasis on AVGs and interactive training programs. In previous scientific reports devoted to research on the feeling of flow during sports activities it has been that there is a moderate relationship between the occurrence of the flow state and the improvement of sports results [48]. Experiencing a state of flow during exercise is also important in promoting PA due to the importance of positive experiences for long-term participation in PA [49]. With regard to computer applications, flow state is considered an important component of the user experience in virtual environments allowing for a better understanding of human–computer interactions [50, 51]. Flow measurement allows to analyse the extent to which users are engaged in specific tasks and is of significance in the design of products such as software, video games and e-learning courses [52]. For AVG creator, flow research can help develop game content so that optimal engagement can occur during gameplay [53]. Flow, while using VR-based AVGs, was considered extremely desirable in the context of patient engagement in rehabilitation [54, 55].

Due to the fact that the border between virtual and real reality is increasingly blurred, research related to the assessment of feelings associated with performing similar forms of PA in different environments is becoming more and more important. Currently, there are no studies in which the authors would simultaneously compare the flow state accompanying identical or similar forms of PA in RL, nIVR and IVR. However, there are reports in which the authors compare two of these environments. According to Fang and Huang [56], the level of perceived flow may be higher when PA is performed in an IVR setting than when similar physical exercises take place in natural conditions. This is confirmed, for example, in the research conducted by Bum et al. [57] regarding playing golf in natural and virtual conditions. In the group of people playing virtual golf, the authors noted a higher level of flow than among people practicing this sport in the real world. Lee and Lee [58] compared the flow of students during football (soccer) classes in primary school using nIVR technology and the traditional form of classes. The results allowed to indicate that students who participated in nIVR classes experienced greater flow than those who participated in traditional ones. There is also scientific evidence that video game users achieve a higher level of flow in IVR compared to nIVR. The immersive sensory and physical affordances of gaming in IVR are more effective in generating a state of flow among players compared to traditional video games [59–61]. However, these observations apply to activities other than PA.

### Correlations between satisfaction and flow

Based on a review of the available literature, it can be concluded that pleasure is positively related to flow [62]. This relationship is also confirmed in the present study. The correlation analysis conducted for the entire study population shows that there are significant positive correlations between PACES and FSS results. This applies to all cycling conditions tested. Their level can be classified as moderate. It is worth emphasizing that the current trial is one of the few conducted in a virtual environment and, to the authors’ knowledge, the first in which these correlations have been demonstrated for the same motor activity simultaneously in RL, nIVR and IVR. The correlation between pleasure and flow in the IVR environment was also studied by Lemmens and von Münchhausen [63]. These authors studied 201 university students playing the popular rhythm game Beat Saber. The authors showed a high positive relationship (*r* = 0.65, *p* < 0.001) between flow experience and satisfaction of users playing AVG in a virtual environment.

## Limitations

The limitations of the conducted research include the relatively small group of subjects, consisting of 40 volunteers, all of whom were students of the Academy of Physical Education. Due to the homogeneity of the sample, it may not be representative of the broader population, and the results are difficult to generalize to a broader demographic group. Additionally, the study's focus on a specific age and education group may limit the applicability of the results to the general population. However, the students' choice resulted from the need to test people who had experience both in outdoor cycling and training on a bicycle ergometer. In addition, we had to be sure that each of the subjects would be able to continuously perform similar physical effort throughout the duration of the experiment, because we assumed that the intensity of physical exercise and the level of fatigue affect the participants' satisfaction and flow.

Another limitation was the exclusion from the study of people sensitive to flashing lights, epilepsy and motion sickness. This may have inadvertently excluded individuals who could provide valuable information on the impact of IVR on recreational cycling. However, this procedure was necessary for the safety of the subjects.

We also consider the limitation of the research to be the fact that nIVR and IVR cycling along a specific route were compared to the experience of traditional cycling, which could take place in various terrain and conditions. The comparison would be optimal if the subjects could compare the same route taken in a virtual and real environment, which is theoretically possible, although difficult to do.

## Summary

The existing scientific evidence on the possibility of using VR to influence the level of pleasure and flow experience during exercise is limited and heterogeneous. The positive impact of the virtual environment on the feelings of PA users was observed mainly in high-immersion VR. However, most studies to date have been focused on nIVR, which indicates a significant gap in the current literature [64]. The research conducted in this study complements the existing evidence base in the field of IVR. Moreover, this is the first attempt to directly compare the feelings about practicing a specific form of PA (cycling) in a real environment and nIVR and IVR. Due to the continuous and dynamic development of VR and related training applications, further research on the experiences and sensations of people undertaking PA using this modern technology is justified in order to identify the factors influencing the attractiveness of this exercise form. This applies to both healthy and clinical populations. Due to the above, it will be possible to optimise recreational and sports exercises as well as rehabilitation programmes in VR and to determine their further development directions. It should be borne in mind that satisfaction and flow are important reasons for undertaking health-promoting PA in a systematic manner. How people feel while exercising determines their future training commitment [1]. Identifying users' preferences regarding forms of PA in VR may make it easier for manufacturers to create attractive training applications and AVGs, which should translate positively into the future behaviour of people using modern technologies during exercise, increasing the likelihood of them systematically undertaking PA.

## Conclusion

1. The form of recreational cycling significantly affects satisfaction and flow related to PA. The most attractive turned out to be physical exercise in IVR, which causes a high level of satisfaction and flow among users. Second place, in this respect, is occupied by typical off-road cycling. A significantly lower level of satisfaction occurs during AF on an stationary bike when the user is exposed to nIVR and when no image is presented (classic indoor training). The last two training conditions also recorded the lowest flow.
2. The questionnaires used in the research (PACES and FFS-2) can be used to assess satisfaction and flow with practicing cycling in nIVR and IVR with regard to young, physically fit adults, because they are characterised by high measurement reliability.
3. A significant positive moderate correlation was found between satisfaction with PA and flow of users practicing various forms of cycling in real-world conditions and virtual reality.

## Practical conclusions and implications for further research

The greater attractiveness of PA and the higher level of flow in IVR compared to the typical form of PA in the field, found on the basis of the conducted research, should prompt reflection by both those involved in planning and promoting PA, as well as the creators of AVGs and multimedia training programmes. Due to the great attractiveness of PA in IVR and the constant, dynamic development of immersive information technologies, virtual training may, in the near future, become not only an important supplement to conventional forms of exercise, but perhaps even an alternative solution. Within the context of the non-obvious and quite unexpected results of the conducted research, it seems reasonable to carry out similar research experiments in which other popular forms of AF that have their counterparts in VR would be taken into account.

## Data Availability

The datasets during and/or analyzed during the current study available from the corresponding author on reasonable request.

## Abbreviations

AVG: Active video game
CAVE: C-Automatic Virtual Environment
FSS: Flow State Scale
HMD: Head-mounted display
IVR: Immersive virtual reality
nIVR: Non-immersive virtual reality
PA: Physical activity
PACES: Physical Activity Enjoyment Scale ()
RL: Real life
VR: Virtual reality
